# The Sins of the Father*Read before the Gross Medical Club at Alden, N. Y.

**Published:** 1913-07

**Authors:** J. Henry Dowd

**Affiliations:** Genito-Urinary Surgeon Sisters’, Mercy and Contagious Hospitals Originator of the Dowd Phosphatometer


### The Sins of the Father.*

BY J. HENRY DOWD, M.D.
Genito-Urinary Surgeon Sisters', Mercy and Contagious Hospitals
Originator of The Dowd Phosphatometer

AT least 99 per cent. of the laity, and a goodly proportion of members of the medical profession, still believe "that the sins of the father," which they interpret to be syphilis and the venereal diseases, "are visited unto the children of the third and fourth generation."

Most careful investigation has been made along lines of this belief and all have arrived at the same conclusion—syphilis, which is the only constitutional disease that can be transmitted as such, never has been positively found to pass the second generation. The same may be said regarding gonorrhea and chancroids, which are looked upon as constituting sins of the father.

Let us go a step farther and assume that tuberculosis may be contracted through abuse of health, and thus be considered a sin of the father, being carried to his children's children, but this we know as next to impossible.

It may therefore be stated quite positively that the blood is in no way a carrier of contagion, or sins of the father, beyond the second generation, and even to the second generation is rarer than is naturally supposed.

One thing is almost a positive fact—in practically almost every case the mother must be syphilitic to produce a syphilitic child.

Syphilis is more rare in women than men. Why? Only the lymph and blood are carriers of the spirocheta. When a man has the initial lesion, patches covered with lymph or abrasions of any kind where blood and lymph are present, he is careful lest he infect another. In the absence of the above there being little or no danger, the female is spared to a great extent.

But let the female become infected, especially in the early stages, and conception take place, is not the almost invariable result abortion; is not this nature's way of protecting the human race from degeneration?

What then are the sins of the father? In speaking of the father, the mother must be included, for is she not a party to the act, carrying the foetus and rearing it to child and manhood?

This question may be answered in one word, taints or traits, which may include anything which enters into the daily life of the human being.

Knowing such to be positive facts, we are face to face with the new science—eugenics—the science for the betterment of the human race, and I might add, their only salvation.

* Read before the Gross Medical Club at Alden, N. Y.

Since time immemorial this science has been applied to domestic animals, by human aid; and in wild state by instinct, or an unseen aid.

But when we come to the human species, the future is allowed to take care of itself; thus we find our asylums full of insane, idiots, inebriates; our prisons full of criminals; tuberculosis and cancer ravaging the race (in practically every case of cancer or tuberculosis it is possible to find a family history of those diseases; they are not transmitted as such, but by a weakened strain); our sanitariums full of neurotics, and homes unhappy, far more than the average person suspects, many of them ending in the divorce court, and all because little or no attention was paid to ancestral stock. "It was a case of love at first sight." How many of these have ended well, have produced children that have been an honor to their parents and humanity. Far be it from me to guess, but the great majority have been ended in the courts of our land. And the future will take care of itself in exactly the way the Bible says, for "like will produce like," and continue to do so, excepting under one consideration: an offspring will partake of characteristics of both parents; thus if we have a pronounced maternal neurasthenic with a father of an exactly opposite type, the child will be what might be termed a halfbreed; although having well pronounced motherly traits, it will partake of the father's sufficiently to raise it far from the mother's condition. (This is simply the Mendelian trait, or theory.)

Other characteristics on the part of the parents must be viewed in a similar manner to that of neurasthenia.

Let us suppose that both parents are above criticism, as far as their family history can show, yet their offspring goes wrong, becomes a drunkard, burglar, murderer, prostitute, developes neuroses or other condition not conductive to good citizenship.

This is easily explained. There are two ways of attaining a fortune, it may be inherited or acquired; there are two ways of possessing characteristics, inherited or acquired.

There can be no denying the fact, a child may be born of parents of unquestionable parental or ancestral taints, but if the child from a babe is reared in a vicious environment it will undoubtedly acquire the viciousness of its surroundings.

Still another example may be stated. Both parental and ancestral history may be as near perfect as it is possible to attain, but the father had gonorrhea when a young man. Gonorrhea is a mixed infection due to gonococci, staph. and streptococci. Of these bacteria the first is known to perish, in 98 per cent. of cases, inside of ten weeks, but they leave behind their allies, and usually deeply buried in the prostatic sinuses, from which they do not make their appearance excepting at the time of coitus. The young man has had no discharge for some time, even the urine, macroscopically, is clear; he marries. Sooner or later the young wife complains of pains, profuse menses, leucorrhea, etc., and an examination reveals a diseased ovary (strepto. or staphylococci can do as much damage to an ovary as to an appendix) ; it must come out.

The pain has more or less racked the nervous system, the removed ovary has added its share, and a neurasthenic has been produced. Later on a child is born. What is it? A neurotic, of course, to a degree according to the condition of the father, who due to the fast life now necessary in its reach for riches may have become a neurotic also. It is at this point it may be admitted that the sin of the father has been visited.

Before offering too adverse criticism regarding eugenics with the statement that sons of clergymen, and even their daughters, have been known to turn out vicious, listen. Do not judge men, or even women, as being perfect from the clothes they wear or the position they occupy. I have personal knowledge of one clergyman with syphilis, two with gonorrhea, several drunkards (all quietly, of course), and some neurasthenics whose children have never seen a well day. It is obvious to remark that the man or women who poses as angelic, yet quietly commits known sins, cannot help but transmit their taint to the offspring.

In conclusion it may be said the world can be made practically perfect, but it will take a few centuries, and none of us will see it, but a start can be made now; it must be in the choice of life partners for your sons or daughters.

Forget pretty dresses, pretty faces, social standing, good salary, neat dresser, popular club or college man; look to family history, not the immediate family, but as Henry Ward Beecher has said, "the grandparents."

If this is done and family history of cancer, tuberculosis, criminality, drunkenness, fast living (sporting lives), marked neurotic conditions as insanity, epilepsy, St. Vitus dance, vicious tempers and even hysteria and pronounced neurasthenia be taken into consideration, and eliminated from both sides, in 100 years a race will have come forth that we can be proud of.

Again I say, "like begets like." Eugenics is the salvation of the human race.

40 North Pearl Street.

